# Actinomycosis

**Published:** 1901-01-12

**Authors:** 


[1] Lancet, Jan. 5.

### ACTINOMYCOSIS.

The series of cases of actinomycosis reported by Mr. Rickman Godlee [1] should put us all on the *qui vive*, and should do much to awaken the profession to the fact that this hitherto little-known disease may be of far more frequent occurrence than would be imagined from the number of cases which have been recorded. It is, he says, a humiliating thought that we are seeing, but not observing, forms of disease which will seem quite commonplace to our successors, if not to ourselves, and, what is worse, that we are treating them wrongly because we do not recognise their true nature. To the clinician the first sight of the fungus is usually obtained in the pus evacuated from an abscess, or in the expectoration, and it is visible to the naked eye as small round granules, sometimes very minute, sometimes larger, oftenest of a pale yellow colour, but sometimes white, which are easily demonstrated by allowing the pus or expectoration to flow down the side of a test tube while it is held up to the light, or to run over a microscopic slide. They have been compared to particles of iodoform, but they are obviously rounded and are not of such a bright yellow colour. One should always be on the *qui vive* and get into the habit of looking at the pus from any abscess of doubtful origin from this point of view, but especially if on opening the abscess the amount of pus is less than was expected, and the finger passes into an indefinite soft mass which bleeds with great freedom. If one of these yellow granules be placed unstained in a little water on a slide, and the cover glass be gently pressed upon it, it will be seen, under a low power, to be made up of rounded masses which are yellowish on the circumference but less coloured in the centre. Under a high power the centre is seen to be made up of a densely-felted mass of threads of mycelium, and the circumference to contain the so-called clubs which, from their radiated arrangement, have given the name to the fungus. In some cases, however, these are not to be seen.

We need not go further into a description of the tumours and ulcers and suppurations set up by this fnngus. For full particulars we must refer our readers to Mr. Godlee's article. All we have to do now is to draw attention to the necessity of looking for the fungus in all suppurations of doubtful origin, and of bearing the disease in mind when considering the nature of obscure tumours. Theimportance of the matter, and the probability that many more cases of this disease might be found if they were carefully looked for, are emphasised by the fact that on the same date as that on which Mr. Godlee published his paper a case of actinomycosis was also published by Dr. Littledale,[2] of Dublin. In this case, as in some of Mr. Godlee's, actinomycosis and tuberculosis co-existed.

[1] Lancet, Jan. 5. [2] Brit. Med. Jour., Jan. 5.

